# Incidence and risk factors for early postoperative stiffness after arthroscopic rotator cuff repair in patients without preoperative stiffness

**DOI:** 10.1038/s41598-022-07123-5

**Published:** 2022-02-24

**Authors:** Chul-Hyun Cho, Ki-Choer Bae, Du-Han Kim

**Affiliations:** Department of Orthopedic Surgery, Keimyung University Dongsan Hospital, Keimyung University School of Medicine, 1035 Dalgubul-ro, Dalseo-gu, Daegu, 42601 South Korea

**Keywords:** Risk factors, Prognosis

## Abstract

The purpose of this study was to investigate the incidence and risk factors of early postoperative stiffness in patients without preoperative stiffness undergoing isolated arthroscopic rotator cuff repair (ARCR). Two hundred seventy-four patients who underwent primary ARCR were included. At 3 months after surgery, criteria for shoulder stiffness was set as follows: (1) passive forward flexion < 120˚, or (2) external rotation at side < 30˚. Patients with preoperative stiffness or who underwent additional procedures were excluded. Patients-related, radiological (muscle atrophy and fatty infiltration), and intraoperative (tear size, repair techniques, number of anchors used, and synovitis scores) risk factors were analyzed. Univariate and multivariate analyses were used to identify risk factors for postoperative stiffness. Thirty-nine of 274 patients (14.2%) who underwent ARCR developed postoperative stiffness. Univariate analyses revealed that early postoperative stiffness was significantly associated with diabetes mellitus (*p* = 0.030). However, radiological and intraoperative factors did not affect postoperative shoulder stiffness (all *p* > 0.05). Multivariate analyses revealed early postoperative stiffness was significantly associated with diabetes mellitus and timing of rehabilitation (*p* = 0.024, *p* = 0.033, respectively). The overall incidence of early postoperative stiffness following isolated ARCR in patients without preoperative stiffness was 14.2%. Diabetes mellitus and timing of rehabilitation were independent risk factors for early postoperative stiffness following ARCR.

## Introduction

Rotator cuff tears are a common clinical problem with multifactorial etiology including degenerative changes and trauma. A commonly accepted and widely used treatment approach for rotator cuff tears involves arthroscopic repair^1–3^. Although most patients experience satisfactory clinical outcomes after arthroscopic rotator cuff repair (ARCR), potential complications have also been reported from a number of studies^4–7^. Among reported complications following ARCR, shoulder stiffness is one of the most common, with an incidence ranging from 2.3 to 28.5%^8–12^. Another frequently reported complication—shoulder stiffness—was more common in the early postoperative period (i.e., within 3 months after surgery) with a range from 11 to 35.4%^8,13,14^. Despite an otherwise successful ARCR, postoperative stiffness may lead to a distressing situation and dissatisfaction for both the patient and surgeon^13^. Therefore, the ability to better assess the risk and prevent postoperative stiffness is crucial, particularly in the early postoperative period.

Various risk factors, such as preoperative shoulder stiffness, female, diabetes mellitus (DM), hypothyroidism, operative technique, additional procedures, prolonged immobilization, and glenohumeral joint (GHJ) synovitis, have been suggested as causes of postoperative stiffness after ARCR^8,14–16^. Despite awareness of these risk factors, few studies have comprehensively analyzed patient-related, radiological, and intraoperative risk factors for early postoperative stiffness after ARCR. In addition, little is known about the incidence of early postoperative stiffness in patients without preoperative stiffness undergoing isolated ARCR.

The purpose of this study was to investigate the incidence and risk factors of early postoperative stiffness in patients without preoperative stiffness undergoing isolated ARCR. We hypothesized that the incidence of early postoperative stiffness would be higher than the previously reported values, and specific risk factors might be related to early postoperative stiffness.

## Methods

This study was approved by the institutional review board of our hospital (KMUDSH IRB No: 2020-07-080). The study was carried out in accordance with the Declaration of Helsinki. All patients signed informed consent forms.

**Figure 1:**
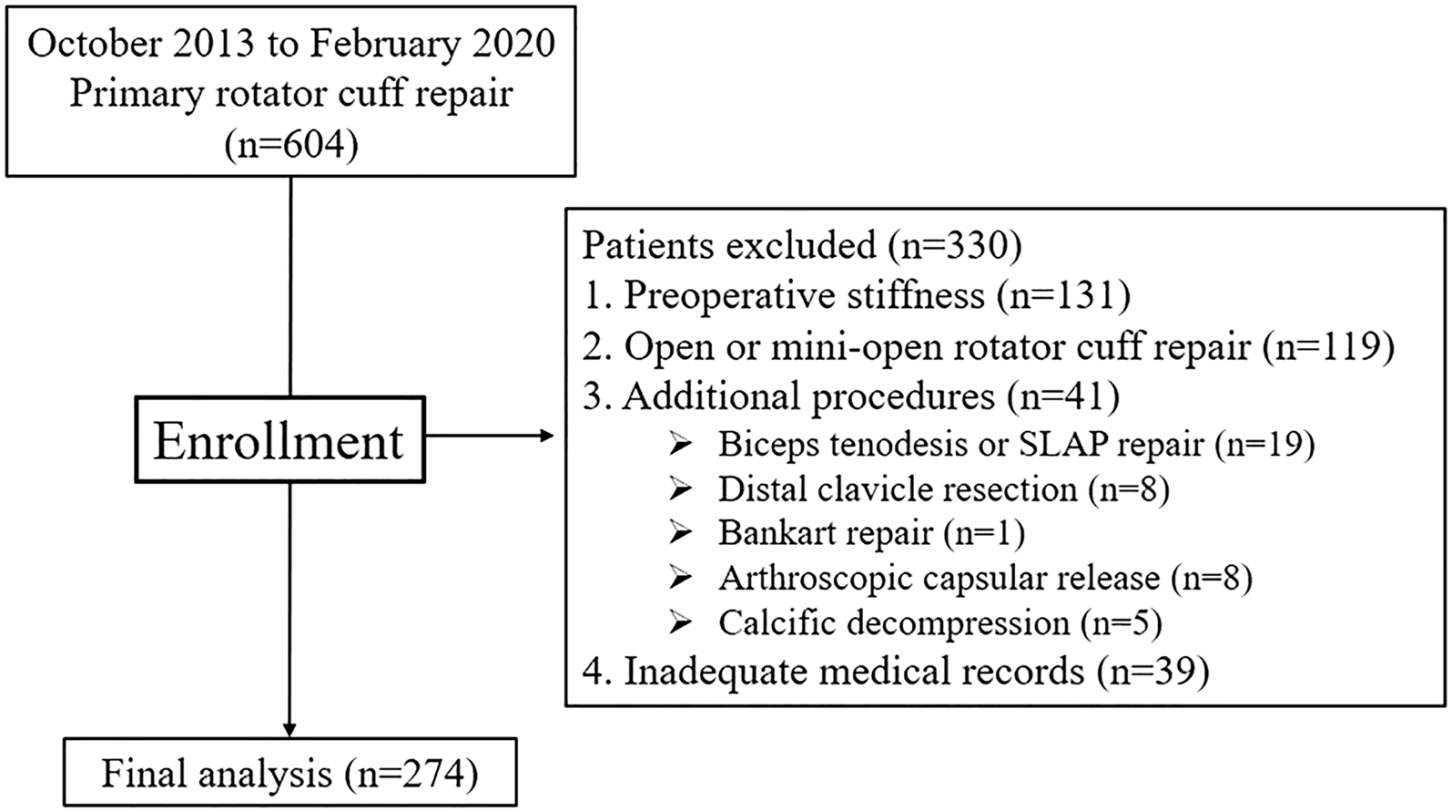
Patient’s chart flow.

Between October 2013 and February 2020, 274 patients who underwent ARCR by a single surgeon were included in this study. Inclusion criteria were as follows: (1) patients with primary ARCR, (2) available medical records, and arthroscopic photos and videos. Exclusion criteria included patients who had: (1) preoperative shoulder stiffness, (2) open or mini-open rotator cuff repair, (3) additional procedures (e.g., biceps tenodesis, labrum repair, stabilization for instability, distal clavicle resection), (4) inadequate medical records (Fig. 1).

### Surgical technique

A single shoulder surgeon performed all procedures with patients in the lateral decubitus position under general anesthesia. A standard arthroscopic GHJ examination through the posterior and anterior portals to evaluate intra-articular pathology was performed. Next, the arthroscope was placed in the subacromial space (SAS), and rotator cuff repair was carried out. The number of anchors and repair techniques (i.e., single row, double row, suture bridge repair) used depended on the tendon mobility, tear size, and pattern. Subacromial decompression for the removal of inflamed bursa tissue and acromioplasty confined to the anterolateral aspect of the acromion was performed in all patients^17^.

### Factors associated with early postoperative shoulder stiffness

1. Patient-related factors
  The following baseline data were collected: age, sex, body mass index (BMI), DM, thyroid disease, hyperlipidemia, duration of symptoms, dominant arm, history of trauma, the intensity of labor, level of sports activity, preoperative University of California, Los Angeles (UCLA) score, preoperative visual analog scale (VAS) pain score, and preoperative American Shoulder and Elbow Surgeons' scale (ASES) score. The intensity of labor was divided into four groups for analysis: (1) heavy labor, (2) light labor (included office work), (3) unemployed, (4) others. The level of sports activity was also divided into four groups: (1) none, (2) overhead sports, (3) contact sports, (4) non-contact sports.
2. Radiological factors
  Plain radiography and MRI (1.5 T scanner, Siemens Magnetom Avanto System; Siemens Medical, Erlangen, Germany) were performed before surgery. Preoperative muscle atrophy was evaluated according to Thomazeau classification for each tendon, and fatty infiltration of muscle was evaluated according to Goutallier classification of each tendon^18,19^.
3. Intraoperative factors
  Tear size was measured intraoperatively using a calibrated probe after debridement of the degenerated tendon edges. The anteroposterior dimension was measured at the lateral edge of the footprint, and medial retraction was estimated as the distance from the apex of the tear to the lateral footprint. According to the grading system proposed by Davis et al.^20^, GHJ synovitis was graded, with total GHJ synovitis scores ranging from 0 to 6. According to the grading system proposed by Jo et al.^21^, SAS synovitis was graded, and total SAS synovitis scores ranged from 0 to 5.

### Evaluation of a range of motion and definition of shoulder stiffness

To evaluate shoulder range of motion (ROM), passive motion in two directions (forward flexion, external rotation at the side) was measured with a goniometer. Forward flexion was measured in degrees between the arm and the thorax with the elbow held straight, and external rotation with 0˚ of shoulder abduction was measured with the elbow in 90˚ of flexion between the thorax and the forearm^22^. An independent research coordinator performed a clinical examination. Criteria for shoulder stiffness was set as follows: (1) passive forward flexion < 120˚, or (2) external rotation at side < 30˚ according to the definition of Oh et al.^22^. Patients were included in the stiffness group if they exhibited either or both of these criteria at 3 months after surgery.

### Postoperative rehabilitation

All patients underwent active ROM of the finger, elbow and hand exercise from the first day after operation. An abduction brace was applied, and the duration was based on the tear size measured at the time of surgery. Patients with small- to medium-sized rotator cuff tears wore the abduction brace for 4 to 6 weeks and were allowed passive ROM of the shoulder from 2 to 4 weeks postoperatively, while patients with large to massive tears wore the brace for 6 weeks and were allowed passive ROM from 4 to 6 weeks postoperatively. Patients began active ROM exercises at 6 weeks after surgery, rotator cuff strengthening exercises at 3 months after surgery, and manual labor and sports activities at 6 months after surgery.

### Statistical analysis

The SPSS statistical package (version 25.0; IBM, Armonk, NY, USA) was used for data analysis. A power analysis indicated that a sample consisting of 230 patients would provide 95% statistical power with an α of 0.05 for medium effect size (f^2^ of 0.15) for multiple linear regression. The independent t-test and chi-square test were used to compare baseline demographics data between both groups. Univariate analysis was used to identify risk factors associated with postoperative stiffness. Significant associations observed in univariate analysis and significant variables reported in the literature were included in the multivariate analysis^8,14,15,23,24^. Statistical significance was accepted for *p*-values of < 0.05.

### Ethics declarations

This study was approved by the Institutional Review Board of Keimyung University Dongsan Hospital (IRB No. 2020-07-080).

### Consent to participate

All patients gave their oral and written consent for their clinical and radiological data to be analyzed and used in this study.

### Consent to publish

All patients gave their oral and written consent for their clinical and radiological data to be published.

## Results

Of the 274 patients treated with primary ARCR, 39 patients (14.2%) developed shoulder stiffness at 3 months after surgery.

### Patient-related factors

There were no significant differences in age, sex, BMI, thyroid disease, hyperlipidemia, duration of symptom, dominant arm, trauma, the intensity of labor, or level of sports activity between the two groups (all *p* > 0.05), however, the prevalence of DM was significantly different between the two groups (*p* = 0.030). DM was observed in 20.5% (8/39) of the stiffness group and 8.9% (21/235) of the non-stiffness group. There were no significant differences in preoperative UCLA score, VAS pain score, ASES score, and the timing of rehabilitation between the two groups (all *p* > 0.05) (Table 1).

**Table 1:** Patient-related factors resulting in early postoperative shoulder stiffness.

	Non-stiff group (n = 235)	Stiff group (n = 39)	*p* value
Age, year	60.3 ± 8.15	62.1 ± 7.2	0.204
Sex, male/female, n	90/145	14/25	0.775
BMI, kg/m^2^	25.0 ± 3.1	25.2 ± 3.0	0.753
Diabetes mellitus, yes/no, n	21/214	8/31	0.030*
Thyroid disease, yes/no, n	29/206	8/31	0.167
Hyperlipidemia, yes/no, n	20/215	4/35	0.759
Duration of symptoms, months	27.0 ± 32.5	26.1 ± 25.6	0.877
Dominant arm. yes/no, n	174/61	32/7	0.284
History of trauma, yes/no, n	46/189	5/34	0.183
**The intensity of labor**			0.361
Heavy Light Unemployed Etc	91 61 80 3	13 9 17 0	
**A level of sports activity**			0.599
No Overhead Contact Non-contact	168 22 6 29	27 2 0 10	
Preoperative UCAL score	17.1 ± 4.8	15.7 ± 4.3	0.090
Preoperative VAS score	5.9 ± 2.3	6.0 ± 2.3	0.778
Preoperative ASES socre	46.8 ± 17.6	44.0 ± 18.4	0.367
Timing of rehabilitation, days	24.1 ± 10.8	27.8 ± 13.3	0.110

*BMI* body mass index, *UCLA* University of California, Los Angeles, *VAS* visual analog scale, *ASES* American Shoulder and Elbow Surgeons’ Scale.

*Statistically significant, *p* < 0.05.

### Radiographic and intraoperative factors

Muscle atrophy greater than grade 2 was observed in 20.4% (48/235) of patients in the non-stiffness group and 33.3% (13/39) of those in the stiffness group, however, this difference was not significant (*p* > 0.05). The grade of fatty infiltration was also not significantly different between the two groups (*p* > 0.05). An analysis of intraoperative factors revealed that there were no significant differences in tear size, repair techniques, number of anchors, total GHJ score, or total SAS score between the two groups (all *p* > 0.05) (Table 2).

**Table 2:** Radiologic and intraoperative factors resulting in early postoperative shoulder stiffness.

	Non-stiff group (n = 235)	Stiff group (n = 39)	*p* value
*Radiologic factors*			
**Muscle atrophy**			0.269
Grade 1 Grade 2 Grade 3	187 40 8	26 13 0	
**Fat infiltration**			0.821
Grade 0 Grade 1–2 Grade 3–4	17 187 31	2 32 5	
*Arthroscopic factors*			
**Tear size**			0.814
Partial Small-medium Large-massive	29 123 83	5 19 15	
**Repair technique**			0.252
Single row Double row Suture bridge	51 101 83	5 18 16	
Number of anchors	2.7 ± 1.1	2.8 ± 0.9	0.648
GHJ total score SAS total score	3.5 ± 1.3 2.0 ± 1.9	3.5 ± 1.4 2.0 ± 1.2	0.889 0.883

*GHJ* glenohumeral joint, *SAS* subacromial space.

*Statistically significant, *p* < 0.05.

### Multivariable regression analysis

Multivariate analyses included age, sex, duration of symptom, DM, thyroid disease, total GHJ score, tear size, and timing of rehabilitation. In the multivariable regression model, DM and timing of rehabilitation were identified as independent risk factors for early postoperative stiffness (*p* = 0.024 and *p* = 0.033, respectively) (Table 3).

**Table 3:** Factors affecting early postoperative stiffness: results of multivariate logistic regression analysis.

Variable	Β	Odds Radio	Unstandardized coefficient beta (95%CI)	*p* value
Sex	−0.098	0.907	0.431–1.908	0.797
Age	0.036	1.036	0.985–1.091	0.171
Symptom duration	0.000	1.000	0.989–1.012	0.956
Diabetes mellitus	1.090	2.975	1.155–7.661	0.024*
Thyroid disease	0.563	1.755	0.709–4.345	0.224
GHJ total score	−0.078	0.925	0.696–1.228	0.588
Tear size	−0.546	0.580	0.291–1.154	0.121
Timing of rehabilitation	0.040	1.041	1.003–1.080	0.033*

Variables that showed a significant relation with postoperative stiffness in univariate analyses and significant variables reported in the literature were included in this multivariate logistic regression analysis.

*β* estimated regression coefficient, *CI* confidence internal, *GHJ* glenohumeral joint.

*Statistically significant, *p* < 0.05.

## Discussion

The most important finding of the present study is that postoperative stiffness at 3 months after isolated ARCR in patients without preoperative stiffness is a common complication with an overall incidence of 14.2% (39/274). Additionally, DM and the timing of rehabilitation were independent risk factors for early postoperative stiffness following ARCR.

Postoperative shoulder stiffness following ARCR may affect functional outcomes and a patient's satisfaction for the procedure. Because of the clinical importance of postoperative shoulder stiffness, an improved understanding of its incidence and a consistent definition is crucial for orthopedic surgeons. In a review of the literature, the incidence of postoperative stiffness following ARCR varies from 2.3 to 28.5%^8–12^. Reasons for this wide range were the potential subjectivity of the criteria, and inconsistent timing of measurements. Several authors defined shoulder stiffness as passive forward flexion of less than 100˚ and external rotation less than 30˚^8,11,25^. Of the authors using this definition, Brislin et al.^25^ reported that 23 of 263 patients (8.7%) had shoulder stiffness 3 months after ARCR, and Parsons et al.^11^ noted that 10 of 43 patients (23.3%) experienced shoulder stiffness 6 to 8 weeks after ARCR. While Tan et al.^8^ reported that 32 of 290 patients (11%) experienced postoperative shoulder stiffness at their 3 months follow-up visit, 25/32 (78.1%) had resolution of stiffness by 9–12 months. Kim et al.^26^ defined shoulder stiffness as forward flexion of less than 140˚ or external rotation with the arm in 90˚ abduction of less than 40˚. They noted that 74 of 209 patients (35.4%) experienced postoperative stiffness within 6 weeks after ARCR. Chung et al.^14^ set the criteria of shoulder stiffness for forward elevation at less than 120˚, external rotation with the arm at the side at less than 30˚, or internal rotation at the back as lower than the third lumbar vertebral level, as previously described by Oh et al.^14,22^. According to these criteria, they reported that postoperative stiffness was observed in 18.8% (54/288) of patients at 3 months after ARCR, 2.8% (8/288) at 6 months, and 6.6% (19/288) at the final follow-up (mean 13.5 months)^14^. However, these previous studies included patients with preoperative stiffness or additional procedures that can affect postoperative stiffness. In the present study, 39 of 274 patients (14.2%) who underwent ARCR developed postoperative stiffness at 3 months after surgery. Unlike previous studies, our study included patients with ARCR only and excluded patients with preoperative stiffness, open or mini-open rotator cuff repair, and any who underwent additional procedure. Stiffness was defined as forward flexion of less than 120˚ and external rotation with the arm at the side of less than 30˚ in our study. These criteria were selected because they are easy to examine in the outpatient clinic. Several studies have included a motion of internal rotation in the definition of stiffness^4,22^. However, we excluded an internal rotation from the diagnostic criteria of postoperative stiffness because we thought that a hand-behind-the-back ROM might not accurately assess active and passive internal rotation of the shoulder^27^.

Although the etiology of stiffness after ARCR might be multifactorial and not completely understood, various risk factors (e.g., female gender, younger in age, DM, preoperative stiffness, hypothyroidism, systemic lupus erythematosus) are reported to be associated with postoperative stiffness^10,15,23,28^. In particular, DM—which is a risk factor for frozen shoulder—has been widely studied for stiffness after ARCR^13–15,23,29^. Although some articles had not found an association between DM and postoperative stiffness^13,14^, several studies have reported that DM could be a potent risk factor for postoperative stiffness^15,23^. Blonna et al.^15^ reported that the overall incidence of postoperative stiffness was 29% (19/65) in patients who underwent ARCR or arthroscopic subacromial decompression. In their study, of the 12 patients who had DM or pre-diabetes conditions, 5 (42%) developed postoperative stiffness (relative risk = 5.7, *p* = 0.03). Burrus et al.^23^ analyzed 232 of 19,229 patients (1.2%) who underwent lysis of adhesions or manipulation under anesthesia after isolated ARCR using the PearlDiver Patients Records Database. They reported that type-1 DM was a significant risk factor (odds ratio = 2.7, *p* < 0.0001). In the present study, it was also noted that DM was independently associated with postoperative stiffness at 3 months; 20.5% (8/39) of DM patients were in the stiffness group compared to 8.9% (21/235) in the non-stiffness group.

The present study revealed that rehabilitation was significantly associated with postoperative stiffness, and previous investigations also have shown that rehabilitation was closely related to postoperative stiffness^24^. Parsons et al.^11^ retrospectively evaluated 43 patients who underwent full-time sling immobilization without formal therapy for 6 weeks after ARCR. They concluded that slower rehabilitation does not result in increased long-term stiffness, but 23% (10/43) patients were determined to have postoperative shoulder stiffness at 6 to 8 weeks after surgery. Koo et al.^10^ performed primary ARCR in 152 patients and patients with risk factors identified in the previous study (i.e., calcific tendonitis, adhesive capsulitis, partial articular surface tendon avulsion type rotator cuff tear, concomitant labral repair, single-tendon cuff repair) were enrolled in a modified rehabilitation protocol that added early overhead closed-chain passive motion exercises. They reported that no patients experienced postoperative stiffness at a mean of 8-month follow-up compared to a control group (7.8%). However, Galatz et al.^30^ found that early motion, even passive motion, may result in devastating consequences. This group reported a high percentage (94.4%) of recurrent defects in patients with early passive rehabilitation after ARCR.

Another potential variable is GHJ synovitis or SAS bursitis^8,31–33^. Tan et al.^8^ reported that the GHJ synovitis score was independently associated with postoperative shoulder stiffness at 3 months after ARCR. Tauro^9^ analyzed 72 patients with rotator cuff tears and concomitant preoperative shoulder stiffness who underwent ARCR and also found that bursitis and articular synovitis were more advanced in the group with severe stiffness. However, scores of GHJ synovitis and SAS bursitis were not significantly different between both groups in the present study.

This study has several limitations. First, it is a retrospective analysis, however, our data were collected prospectively by a single research coordinator. The second limitation is the short follow-up. Patients were followed up for 3 months. But, this decision was made since our primary goal was to analyze the incidence and risk factors of early postoperative stiffness. Third, patients in this study were not routinely assessed with postoperative imaging for the presence of cuff re-tear. Nevertheless, the strength of this study is that patients treated with isolated ARCR were included, and patients who underwent additional procedures or preoperative stiffness were excluded. Furthermore, the investigation was performed in a homogenous group of patients who underwent ARCR by a single surgeon, with a relatively large number of cases (274 patients) with various potential risk factors.

## Conclusion

The overall incidence of early postoperative stiffness following isolated ARCR in patients without preoperative stiffness was 14.2%. DM and timing of rehabilitation were identified as independent risk factors for early postoperative stiffness after ARCR. These findings may allow surgeons to adjust postoperative management in an attempt to prevent early postoperative stiffness. Additionally, surgeons may consider discussing these risk factors with patients before surgery.

## Data Availability

All data and materials used in this work are available.
